# News and Events of the Week

**Published:** 1910-08-27

**Authors:** 


### News and Events of the Week.

Mr. John Harris, C.C., of Gordon Square, W.C., has bequeathed £500 to the Jewish Hospital and Home for Incurables; £200 each to the London Hospital and the Victoria Park Hospital; and £1,000 to the Jewish Board of Guardians upon trust for a "John Harris Fund," to apply the income in assisting persons of the Jewish faith after leaving convalescent homes, infirmaries, hospitals, or asylums.

The winter session of the Middlesex Medical School will open on Monday, October 3, at 3 p.m., when Mr. W. Salmon Nowell will give an address, after which the prizes gained during the previous year will be distributed. The annual dinner of the past and present students and their friends will take place at the Trocadero the same evening, at 7 o'clock precisely, Mr. Thomas H. Kellock in the chair.

At the International Congress on School Hygiene, which was formally opened at the Sorbonne on Tuesday, August 2, the question of physical culture was discussed. Dr. Kerr, Medical Officer of the London County Council, speaking with regard to the medical inspection of school children, said he considered it almost valueless, unless accompanied by medical treatment. The various sections afterwards discussed the question of open-air schools and a variety of other subjects, such as playgrounds, sanitary records, parasitic diseases, schools for the mentally abnormal, and tests for hearing.

Some time ago, according to *L'Aurore*, while making excavations in the cellars of the Taverne du Panthéon, workmen unearthed a number of coins wrapped up in a garment. The coins turned out to be of no great value to collectors, but the garment was found to be made of human skin, a fact vouched for by a professor at the Museum, who, however, attached no great importance to the discovery. In "L'Histoire de Montgaillard" it is stated that human skin was tanned at Meudon, and that this workshop produced excellent examples of the industry. The Duc d'Orléans (Egalité) possessed a pair of trousers made of human skin. Well-developed corpses of executed felons were flayed and their skin tanned with the greatest care. A man's hide yields leather of a consistency and of a degree of beauty superior to that of the chamois. A woman's skin is less solid on account of the softness of the tissue.

Sir Francis Seymour Haden, F.R.C.S., of Woodcote Manor, Bramdean, Alresford, Hants, founder and President of the Royal Society of Painter-Etchers, who died on June 1, aged ninety-two, left estate of the gross value of £32,138 6s. 11d., of which the net personalty has been sworn at £30,885 10s. 7d. The testator left £300 to the treasurer for the time being of the Royal Society of Painter-Etchers, to assist the Society in coping with difficulties which may arise in future; the decollated head of Monmouth (probably Dobson) to the National Portrait Gallery, "this bequest only to take effect if Lionel Cust inspects and approves as genuine the picture before my death"; otherwise this picture is to devolve in the testator's family as an heirloom. The testator especially directs that his executors are under no circumstances to allow the control of the plates of his etchings to pass out of their hands, and they are to destroy such plates when they shall consider that they no longer give clear impressions.

London-Australia Circular Ticket.—The P. and O. Company is now prepared to issue a circular ticket from London to Australian ports, which will entitle the holder to make the outward journey by the P. and O. Branch Service via the Cape, returning by P. and O. mail steamer via Suez, or *vice versa.* In respect of this circular ticket, half the combined return fares will be payable, plus the surtax of 10 per cent. The cooking, accommodation, and service on board the branch line steamers is of a superior character, and the combined ticket will afford greater variety of route and a wider choice of season than if the journey were made in both directions via Suez.

The preliminary programme of the twenty-fifth Congress of the Royal Sanitary Institute, to be held in Brighton from September 5 to 10, has now been issued. The President of the Congress is the Hon. Sir John A. Cockburn, K.C.M.G., M.D. Dr. A. Newsholme, D.P.H., F.R.C.P. (principal medical officer, Local Government Board), will lecture to the Congress on "The Natural Importance of Child Mortality." Dr. Alex. Hill, M.A., F.R.C.S., J.P., will deliver the popular lecture on "The Bricks with which the Body is Built." Excursions to places of interest in connection with sanitation, conversazione, garden party, visits to Arundel Castle and the Isle of Wight, and other entertainments will be arranged for those attending the Congress. Over 200 authorities, including a number of county councils and county boroughs, have already appointed delegates to the Congress, and as there are also over 3,800 members and associates in the Institute, there will probably be a large attendance in addition to the local members of the Congress. In connection with the Congress a health exhibition of apparatus and appliances relating to health and domestic use will be held as a practical illustration of the application and carrying out of the principles and methods discussed at the meetings; it not only serves this purpose, but also an important one in diffusing sanitary knowledge among a large class who do not attend the other meetings of the Congress. The Congress will include general addresses and lectures. Two section meetings for two days each, dealing with: Section I., "Sanitary Science and Preventive Medicine," President, Professor E. W. Hope, M.D., D.Sc., Medical Officer of Health, City and Port of Liverpool; Section II., "Engineering and Architecture," President, Henry Rofe, M.Inst.C.E., F.G.S. In addition there will be the following eight special conferences: "Municipal Representatives," presided over by the Worshipful the Mayor of Brighton (Councillor Edward Geere); "Port Sanitary Authorities," presided over by William H. Williamson, Chairman Port of London Sanitary Authority; "Medical Officers of Health," presided over by Edward Sergeant, L.S.Sc., L.R.C.P., M.R.C.S., Medical Officer of Health, Lancashire County Council; "Engineers and Surveyors to County and other Sanitary Authorities," presided over by P. H. Palmer, M.Inst.C.E., borough engineer, Hastings; "Veterinary Inspectors," presided over by W. Hunting, F.R.C.V.S.; "Sanitary Inspectors," presided over by W. G. Cooper, chief sanitary inspector, Bournemouth; "Women on Hygiene," presided over by the Countess of Chichester; "Hygiene of Childhood," presided over by Sir William J. Collins, M.P., D.L., J.P., M.D., F.R.C.S. The local arrangements are in the hands of an influential local committee, presided over by the Worshipful the Mayor (Councillor Edward Geere), with Duncan Forbes, B.Sc., M.D., D.P.H., as local hon. secretary. A large number of papers on various subjects will be brought forward for discussion.

THE CHOLERA.

Many fresh cases of cholera are reported from Italy from the affected districts of Bari and Foggia. No case has been detected in any other locality beyond those already known, with the exception of one suspected case at Bari, the result of a bacteriological examination regarding which is awaited. The Ministry of the Interior is continuing to send isolation tents, drugs, and other requisites to the affected localities. The Red Cross has also sent two parties of trained nurses to Trani and Barletta.

The Local Government Board has this week issued a circular letter to port sanitary authorities and certain riparian sanitary authorities, in which attention is drawn to the fact that cholera is again seriously epidemic in Russia, particularly in the St. Petersburg district and at Cronstadt and other Russian ports. The sanitary authorities of British ports trading with Russia, the letter proceeds, should be on their guard against the importation of cholera into their districts by vessels coming from places where the disease has appeared or is likely to appear. In this connection it is essential that the medical officers of health of such British ports should endeavour to keep themselves informed as to the spread of the present outbreak of cholera, and especially as to the continuance of the disease in ports where it now exists and its appearance in other ports not yet known to be affected by it. The statement which the Board issues weekly to the medical officers of health of port and riparian sanitary authorities, and which contains information as to such cholera occurrences as have come under the Board's notice, will be of assistance in this direction. On September 9, 1907, the Board issued a revised general order relating to cholera, yellow fever, and plague on ships arriving from foreign ports. The Board relies on the port and riparian sanitary authorities taking all necessary steps under that order to prevent the introduction of cholera into this country. Special attention should be paid to ships bringing aliens from Russia to British ports.

---

The Local Government Board has sent the following circular letter to the clerks of metropolitan borough councils :—

"I am directed by the Local Government Board to state that it has had under consideration the question of facilitating the prompt use of diphtheria antitoxin in the case of persons who may be attacked by diphtheria or exposed to the infection of the disease, and with that object it has made an order, under Section 77 of the Public Health (London) Act, 1891, sanctioning the provision by the councils of metropolitan boroughs of a temporary supply of diphtheria antitoxin and of medical attendance in connection therewith. Copies of the order are forwarded herewith.

"The arrangements with respect to the keeping, distribution, and use of the diphtheria antitoxin are to be made in accordance with the advice of the medical officer of health, and, having regard to the importance of the matter, I am to suggest that at an early date the council should consider, with its medical officer of health, what arrangements should be made for the provision of antitoxin, and for its use both as a prophylactic and a curative agency.

"In cases in which a patient is under the care of a medical practitioner, the latter should, in ordinary circumstances, be the person to administer the antitoxin. In considering the matter the council should take into special account the possible requirements of the Poor Law medical officers, as it appears to the Board to be desirable, where the council avails itself of the powers now given it, that the Poor Law medical officers should be able to obtain from the store of the council the antitoxin which they may require from time to time for use in connection with patients under their care.

"To prevent misapprehension it should be observed that the free provision of diphtheria antitoxin which is authorised by the Order must not be regarded as a substitute for removal to hospital of a patient suffering from diphtheria, nor as implying that the patient to whom the antitoxin has been administered may properly be retained for treatment at home, unless means are available for his efficient isolation to the satisfaction of the medical officer of health.

"The prompt administration of antitoxin before the patient is removed to hospital may, especially if delay in removal is inevitable, go far towards preventing the attack of diphtheria from being fatal.

"By Article II. of the Order the duties of the medical officer of health are to be deemed to extend to and to include all action by the medical officer of health in the execution of the Order; and Article III. provides for the payment by the council to the medical officer of health of reasonable compensation for such action taken by him.

"The arrangements which the council may make for the supply of diphtheria antitoxin should be brought to the knowledge of all medical practitioners practising within their jurisdiction; and in any circular letter which the council sends out to medical practitioners it is desirable that emphasis should be laid on the importance of prompt treatment by antitoxin, and of the saving of life which may thereby be effected.

---

Messrs. J. and A. Churchill, of 7 Great Marlborough Street, announce that the circular sent annually to each member of the profession in connection with the *Medical Directory* will be posted on September 1. It will be recollected that this is a month later than was the case last year. Though the change involves considerable extra pressure of work, it is likely to be greatly appreciated, and it has proved to be an advantage to avoid making applications for returns during the holiday month of August. It will greatly facilitate the work of compilation if practitioners will be so good as to return the circular without any delay. As is now usual, the *Medical Directory* will be published before Christmas. The Climatological and Balneological section by Mr. N. H. Forbes, F.R.C.S.Edin., has been extended to include Continental as well as British spas and health resorts.

